# Older Adult Use and Outcomes in a Digital Musculoskeletal (MSK) Program, by Generation

**DOI:** 10.3389/fdgth.2021.693170

**Published:** 2021-08-03

**Authors:** Grace Wang, Jeannie F. Bailey, Manshu Yang, Jeffrey Krauss

**Affiliations:** ^1^Director of Clinical Research, Hinge Health, Inc., San Francisco, CA, United States; ^2^Department of Orthopaedic Surgery, University of California, San Francisco, San Francisco, CA, United States; ^3^Department of Psychology, University of Rhode Island, Kingston, RI, United States; ^4^Hinge Health, Inc., San Francisco, CA, United States; ^5^Department of Orthopedics (Physical Medicine and Rehabilitation Division), Stanford University, Stanford, CA, United States

**Keywords:** telemedicine, aged, engagement, musculoskeletal pain, depression, anxiety, digital technology, utilization

## Abstract

**Objective:** We investigated use and clinical outcomes in a digital musculoskeletal (MSK) program, by generation.

**Method:** This longitudinal study uses retrospective data collected online or by app. The study included adults with 12 or more weeks of pain who took part in a digital MSK program. We compared Gen Z and Millennials, Gen X, working age Baby Boomers, and retiree age Baby Boomer and Silent Generation. Program use outcomes were program start, program completion, and number of exercises, educational articles, and messages to coaches. Clinical outcomes were changes in pain, depression, and anxiety from baseline to 12 weeks. We calculated descriptive statistics and conducted adjusted regression models.

**Results:** Odds of starting the program were significantly higher for Gen Xers (OR: 1.12) and working age Baby Boomers (OR: 1.37) vs. Gen Zers and Millennials. Compared to Gen Zers and Millennials, we observed significantly higher odds of program completion among Gen Xers (OR: 1.62), working age Baby Boomers (OR: 2.24), and retirees (OR: 2.36). Compared to Gen Zers and Millennials, retirees had 19 more exercise sessions (IRR: 1.69), accessed 11 more articles (IRR: 1.84), and sent 4 more messages to coaches (IRR: 1.26). Compared to Gen Z and Millennials, we observed no significant differences in change in pain for Gen Xers, working age Baby Boomers, or retirees.

**Conclusions:** Adults from multiple generations took part in a digital MSK program. Findings suggest that older generations used a digital MSK program more than younger generations, but had similar pain outcomes.

## Introduction

Chronic musculoskeletal (MSK) pain is a leading cause of disability and cost in the United States, especially among older adults. Prevalence and incidence rates in the United States of osteoarthritis, back and neck pain, and other MSK disorders are among the highest in the world (1). In 2018, 134.5 million adults in the United States reported MSK conditions with older adults experiencing higher prevalence rates of MSK conditions and limitations compared to younger adults (2, 3). Furthermore, chronic MSK pain often occurs together with depression and anxiety (4). Pain makes it more challenging to identify depression and anxiety and can exacerbate depression and anxiety symptoms. Depression and anxiety can also increase pain severity, the experience of pain, and the pain duration (5–8).

To prevent and manage MSK pain and associated comorbidities, clinical guidelines recommend evidence-based exercises, education, and additional supports (9, 10). Reviews have concluded that exercise therapy vs. usual care offered pain reduction, reduced depression severity, and improved quality of life (11, 12). Pain neuroscience education can further enhance these benefits (13).

Digital health approaches can facilitate access to these types of conservative therapies by providing interactive tools, connecting users with health teams and offering choices for how, when, and where to access care (14). A meta-analysis of four studies of good methodological quality showed that digital MSK programs significantly improved knee osteoarthritis pain (15). Another review of 8 RCTs of moderate quality found that digital health improved low back pain intensity and disability (16).

We must ensure that digital MSK programs meet the needs of a growing older adult population with MSK conditions. But, to date, no studies have examined whether the use and effectiveness of digital MSK programs differs by generation. Studies have shown that older generations use general technology and digital health technology, but to a lesser extent compared to younger generations (17–20). Further, the effectiveness for older populations remains uncertain (21).

In summary, gaps remain in our understanding about digital MSK program use and outcomes between generations. Thus, we sought to address two objectives. Our primary objective was to examine differences in digital MSK program use between generations. Our secondary objective was to examine differences in digital MSK program outcomes between generations. Better understanding about program use and outcomes by generation will allow us to make program improvements that meet the various needs and desires of a range of users.

## Method

### Study Design

We conducted a longitudinal study using retrospective data collected from participants of a digital MSK program.

### Intervention

The digital MSK program was offered as a benefit to employees and dependents of participating employers. We recruited through email, workplace posters or presentations, and mailings. Those interested in the program registered online by creating a member profile and completing a baseline questionnaire.

After registering, we reviewed the baseline questionnaires to ensure that participants met the following program criteria: age 18 or older; pain in the low back, knee, shoulder, hip, or neck; baseline visual analog scale (VAS) pain score >0; pain lasted for at least 12 weeks; and member covered by employer's health plan. Exclusion criteria were signs of fracture, joint instability, infection, cancer, and cauda equina syndrome.

All accepted participants received tablet computers with a program app and wearable motion sensors (InvenSense MPU-6050, TDK Electronics, Tokyo, Japan). These materials enabled members to receive technology-guided exercise therapy sessions, coaching, and education for chronic pain. To facilitate exercise sessions, animations and videos within the app demonstrated how to perform light-intensity stretching and strengthening exercises. The app and sensors displayed body position of the participant in real-time while completing exercises and indicated to participants whether they were within the appropriate range of movement.

In addition, a personal health coach communicated with participants via text message, email, or in-app messaging. The program offered participants unlimited text and email messages and up to three phone calls with coaches. Participants could also take part in discussion forums with 20–30 other participants. Finally, participants received educational resources covering their condition and treatment options, as well as behavior change topics, such as catastrophizing, coping methods, and fear avoidance.

Overall, participants were encouraged to complete at least three sensor-guided exercise sessions per week, read at least two education papers per week, and log symptoms twice per week. Participants were also encouraged to engage in at least three aerobic exercise activities per week.

### Study Population

In addition to meeting program criteria, this study applied the following inclusion criteria: registered between February 2017 and April 2020 and 12 or more weeks had passed from the time of registration, had registered for only one pathway (i.e., back, knee, shoulder, hip, or neck), had complete baseline data, and provided informed consent through waiver of written documentation.

### Data Collection

Data were collected online or through the program app at baseline during registration and 12 weeks later.

### Variables

We organized variables around Andersen's model of health service use (22) (Supplementary Figure 1). The model shows that contextual factors (i.e., system, environment) and individual (i.e., predisposing, enabling, and need) factors explain service use factors. These factors, in turn, influence perceived and evaluated health outcomes.

The predisposing factor of participant generation was the independent variable of interest and defined as Gen Z or Millenial (born between 1981 and 1999), Gen X (born between 1965 and 1980), working age Baby Boomer (born before 1964 and under age 65), and retiree age Baby Boomer or Silent Generation (age 65 or older) (23). The rationale for distinguishing working age from retiree age Baby Boomers is retirees may have more time to engage in a digital MSK program or self-care generally.

For our primary study objective about digital MSK program use, we focused on five service use outcomes: program start (i.e., completing one exercise session or accessing one educational paper after registering); program completion (i.e., completing exercise sessions or accessing education articles between program weeks 9 and 12); total exercise sessions by program week 12; total education articles read by program week 12; and total number of member-initiated messages to coaches by program week 12.

For our secondary study objective about digital MSK program outcomes, we focused on three measures captured for each individual participant. Change in pain was pain scores at baseline minus pain scores at 12 weeks. Baseline and 12 weeks pain scores were based on responses to the question “Over the past 24 h, how bad was your [back/knee/shoulder/hip/neck] pain?” from 0 (none) to 100 (worst imaginable) presented on a horizontal visual analog scale. We also examined change in depression or anxiety by 12 weeks among the subgroup with moderate or severe depression or anxiety at baseline. Change in anxiety (no/yes) was defined as reported moderate or severe anxiety at baseline and reported no moderate or severe anxiety at 12 weeks. Moderate or severe anxiety was a score of 10 or higher on the Generalized Anxiety Disorder 7-item scale (GAD-7). Change in depression (no/yes) was defined as reported moderate or severe depression at baseline and reported no moderate or severe depression at 12 weeks. Moderate or severe depression was a score of 10 or higher on the Patient Health Questionnaire 9-item scale (PHQ-9). The GAD-7 and the PHQ-9 with cutoffs at 10 points have been shown to have acceptable performance for identifying anxiety and depression (24–26).

Covariates included contextual (e.g., state of residence), predisposing (e.g., gender, exercise frequency per week [ <1 h, 1–2.5 h, more than 2.5 h]), and need (e.g., program pathway and baseline measures of pain, anxiety, depression, and body mass index categories [underweight, normal, overweight, obese]) factors.

### Statistical Analysis

To characterize the population, we conducted descriptive analyses (e.g., means, frequencies) for predisposing and need factors, by generation. We examined differences using chi-square tests for categorical variables and one-way ANOVA for continuous variables. We conducted unadjusted and adjusted regression analyses, per protocol. For the primary objective, logistic regression was conducted for binary outcomes, including program start and completion. Generalized linear models (Poisson regression) was used among program starters for outcome variables representing counts, including total number of exercise sessions, articles and messages. Models were adjusted for contextual (e.g., state of residence), predisposing (e.g., gender, exercise frequency), and need (e.g., program pathway and baseline pain, anxiety, depression, and BMI) factors.

For the secondary objective, linear regression was conducted for the continuous change in pain outcome. Models were adjusted for contextual (e.g., state of residence), predisposing (e.g., gender, exercise frequency), and need (e.g., program pathway and baseline anxiety, depression, and BMI) factors. Logistic regression was conducted for binary outcomes, including change in anxiety and depression by week 12. This model controlled for contextual (e.g., state of residence), predisposing (e.g., gender, exercise frequency), and need (e.g., program pathway and baseline pain and BMI) factors. All analyses were performed using STATA statistical computing software.

The study was approved by the Western Institutional Review Board and complied with all ethical regulations.

## Results

The digital MSK program registered 13,535 Gen Zers or Millennials (mean age 31.32, SD 4.33, median: 32), 16,982 Gen Xers (mean age 46.15, SD 4.68, median: 46), 9,262 working age Baby Boomers (mean age: 58.70, SD 2.90, median: 58), and 1,462 retiree age Baby Boomers or Silent Generation members (mean age: 68.55, SD 4.17, median: 67). Table 1 compares the characteristics of the different generations who registered for the program. Differences between generations were statistically significant for all variables. Compared to younger generations, a smaller percentage of the retiree age generation was female, exercised <1 h, was in the back pathway, and reported moderate to severe anxiety or depression. The retiree generation also had lower baseline pain than younger generations.

**Table 1:** Description of members who registered for the program.

**Factor**	**Variable**	**Gen Z or Millennial** **(*****n*** **=** **13,535)**		**Gen X (*****n*** **=** **16,982)**		**Working age Baby Boomer (*****n*** **=** **9,262)**		**Retiree age Baby Boomer and Silent Generation (*****n*** **=** **1,462)**		
		**Percent or mean**	**Sd**	**Percent or mean**	**Sd**	**Percent or mean**	**Sd**	**Percent or mean**	**Sd**	***P*-value**
**Predisposing**										
	**Gender (%)**									*p* <0.001
	Female	50.35	50.00	54.07	49.84	53.98	49.84	44.73	49.74	
	Male	44.29	49.67	41.56	49.28	41.09	49.20	45.14	49.78	
	Other or unspecified	5.36	22.53	4.38	20.45	4.92	21.64	10.12	30.17	
	**Weekly exercise (%)**									*p* <0.001
	<1 h	35.30	47.79	41.34	49.25	41.02	49.19	35.02	47.72	
	1–2.5 h	40.80	49.15	39.25	48.83	38.66	48.70	39.81	48.97	
	More than 2.5 hours	23.90	42.65	19.40	39.55	20.32	40.24	25.17	43.41	
**Need**										
	**Pathway (%)**									*p* <0.001
	Back	66.46	47.22	60.53	48.88	50.05	50.00	49.45	50.01	
	Hip	4.53	20.79	6.81	25.19	8.11	27.30	11.29	31.65	
	Knee	27.16	44.48	31.51	46.46	40.62	49.11	37.76	48.49	
	Neck	0.64	7.99	0.38	6.18	0.30	5.49	0.41	6.40	
	Shoulder	1.21	10.94	0.77	8.72	0.92	9.54	1.09	10.41	
	**BMI categories (%)**									*p* <0.001
	Underweight	1.15	10.64	0.63	7.91	0.73	8.54	0.82	9.03	
	Normal	31.56	46.48	18.55	38.87	18.55	38.87	22.91	42.04	
	Overweight	30.08	45.86	30.55	46.06	34.19	47.44	38.44	48.66	
	Obese	37.22	48.34	50.27	50.00	46.52	49.88	37.82	48.51	
	**Baseline pain (mean)**	46.54	21.85	48.15	22.44	47.83	22.72	46.28	22.91	*p* <0.001
	**Moderate or severe anxiety at baseline (%)**	32.88	46.98	23.65	42.49	15.95	36.61	12.11	32.63	*p* <0.001
	**Moderate or severe depression at baseline (%)**	26.52	44.14	20.24	40.18	14.93	35.64	11.63	32.07	*p* <0.001

### Differences in Digital MSK Program Use Between Generations

We examined 5 digital MSK program use outcomes: program start, program completion, and total number of exercise sessions, educational articles, and coach messages. Table 2 shows differences between generations on program start. Out of registrants, 86.17% of Gen Z and Millenials started the program vs. 87.56% of Gen Xers, 90.01% of working age Baby Boomers, and 87.00% of retiree age Baby Boomer and the Silent Generation. In adjusted models, we find that the odds of starting the program were significantly higher for Gen Xers (OR: 1.12, 95% CI: 1.04, 1.20) and working age Baby Boomers (OR: 1.37, 95% CI: 1.25, 1.49) compared to the Gen Z and Millennial group. We detected no statistically significant differences in odds of starting between the retiree age generation vs. the Gen Z and Millennial generation (OR: 1.02, 95% CI: 0.87, 1.20).

**Table 2:** Program start and completion outcomes, by generation.

**Outcome**	**Generation**	**Percent (%)**	**Unadjusted model**			**Adjusted model**		
			**OR**	**95% CI**		**OR**	**95% CI**	
Program start (*n* = number registered)	Gen Z and Millennial (*n* = 13,535)	86.17	Ref			Ref		
	Gen X (*n* = 16,982)	87.56	1.13	1.06	1.21	1.12	1.04	1.20
	Working age Baby Boomer (*n* = 9,262)	90.01	1.45	1.33	1.57	1.37	1.25	1.49
	Retiree age Baby Boomer and Silent Generation (*n* = 1,462)	87.00	1.07	0.92	1.26	1.02	0.87	1.20
Program completion (*n* = number starting the program)	Gen Z and Millennial (*n* = 11,663)	66.91	Ref			Ref		
	Gen X (*n* = 14,870)	75.51	1.52	1.44	1.61	1.62	1.53	1.71
	Working age Baby Boomer (*n* = 8,337)	81.53	2.18	2.04	2.33	2.24	2.09	2.40
	Retiree age Baby Boomer and Silent Generation (*n* = 1,272)	83.02	2.42	2.08	2.81	2.36	2.02	2.75

Table 2 also presents program completion, by generation. Among the members who started the program, 66.91% of Gen Zers and Millennials completed the program compared to 75.51% of Gen Xers, 81.53% of working age Baby Boomers, and 83.02% of retiree age Baby Boomers and the Silent Generation. Compared to Gen Z and Millenials, we observed significantly higher odds of program completion among Gen Xers (OR: 1.62, 95% CI 1.53, 1.71), working age Baby Boomers (OR: 2.24, 95% CI: 2.09, 2.40), and retiree age generations (OR: 2.36, 95% CI: 2.02, 2.75) in adjusted models.

Generation was significantly associated with number of exercise sessions, educational articles, and coaches messages among those who started the program. Compared to Gen Z and Millennials, the retiree age generation had an average of 19 more exercise sessions (adjusted IRR: 1.69; 95% CI: 1.61, 1.71), accessed 11 more articles (adjusted IRR: 1.84; 95% CI: 1.76, 1.93), and sent 4 more messages to coaches (IRR: 1.26; 95% CI: 1.19, 1.32) by week 12 (Table 3).

**Table 3:** Program engagement outcomes, by generation.

**Outcome**	**Generation**	**Descriptive result (mean)**	**Unadjusted model**			**Adjusted model**		
			**IRR**	**95% CI**		**IRR**	**95% CI**	
Number of exercise sessions	Gen Z and Millennial	25.86	Ref			Ref		
	Gen X	33.35	1.29	1.26	1.32	1.34	1.31	1.36
	Working age Baby Boomer	41.20	1.59	1.56	1.63	1.62	1.58	1.66
	Retiree age Baby Boomer and Silent Generation	45.26	1.75	1.67	1.83	1.69	1.61	1.76
Number of educational articles	Gen Z and Millennial	12.57	Ref			Ref		
	Gen X	17.61	1.40	1.37	1.43	1.41	1.38	1.45
	Working age Baby Boomer	22.85	1.82	1.77	1.86	1.80	1.76	1.85
	Retiree age Baby Boomer and Silent Generation	23.73	1.89	1.80	1.98	1.84	1.76	1.93
Number of messages to coaches	Gen Z and Millennial	16.43	Ref			Ref		
	Gen X	19.35	1.18	1.15	1.21	1.17	1.14	1.19
	Working age Baby Boomer	21.17	1.29	1.26	1.32	1.28	1.24	1.31
	Retiree age Baby Boomer and Silent Generation	20.86	1.27	1.20	1.34	1.26	1.19	1.32

### Differences in Digital MSK Program Outcomes Between Generations

Average pain scores decreased 27.13 points for Gen Z and Millennials, 28.21 points for Gen X, 27.28 points for working age Baby Boomers, and 25.60 points for retiree age Baby Boomer and Silent Generation. Compared to Gen Z and Millennials, we observed no statistically significant differences in change in pain for Gen Xers, working age Baby Boomers, or the retiree age generation in adjusted models (Table 4).

**Table 4:** Change in clinical outcomes, by generation.

**Outcome**	**Generation (*n* = people with pre and post scores)**	**Descriptive**		**Unadjusted model**			**Adjusted model**		
		**Mean**	**(SD)**	**Beta**	**95% CI**		**Beta**	**95% CI**	
Change in pain score	Gen Z and Millennial (*n* = 4,000)	−27.13	(23.39)	Ref			Ref		
	Gen X (*n* = 6,861)	−28.21	(23.60)	−1.08	−2.00	−0.17	−0.85	−1.77	0.07
	Working age Baby Boomer (*n* = 4,607)	−27.28	(23.39)	−0.15	−1.14	0.84	−0.39	−1.40	0.62
	Retiree age Baby Boomer and Silent Generation (*n* = 739)	−25.60	(23.09)	1.53	−0.29	3.34	0.46	−1.37	2.29
**Outcome**	**Generation (*****n*** **=** **number with moderate or severe symptoms at baseline)**	**Percent with change by week 12 (%)**		**Unadjusted model**			**Adjusted model**		
				**OR**	**95% CI**		**OR**	**95% CI**	
Improvement in moderate or severe anxiety at baseline to 12 weeks	Gen Z and Millennial (*n* = 1,308)	79.28		Ref			Ref		
	Gen X (*n* = 1,603)	81.16		1.13	0.94	1.35	1.17	0.97	1.42
	Working age Baby Boomer (*n* = 714)	87.82		1.88	1.45	2.45	2.05	1.56	2.69
	Retiree age Baby Boomer and Silent Generation (*n* = 81)	91.36		2.76	1.26	6.07	2.71	1.19	6.20
Improvement in moderate or severe depression at baseline to 12 weeks	Gen Z and Millennial (*n* = 967)	78.39		Ref			Ref		
	Gen X (*n* = 1,304)	77.76		0.96	0.79	1.18	1.05	0.85	1.30
	Working age Baby Boomer (*n* = 647)	81.14		1.19	0.92	1.52	1.31	1.01	1.71
	Retiree age Baby Boomer and Silent Generation (*n* = 69)	85.51		1.63	0.82	3.24	1.47	0.72	2.98

Compared to baseline, 79.28% of Gen Zers and Millennials were no longer reporting moderate to severe anxiety at 12 weeks vs. 81.16% of Gen Xers, 87.82% of working age Baby Boomers, and 91.36% of retiree age Baby Boomers and Silent Generation adults. Compared to Gen Zers and Millennials, working age Baby Boomers (OR: 2.05, 95% CI: 1.56, 2.69) and retiree age Baby Boomers and Silent Generation (OR: 2.71, 95% CI: 1.19, 6.20) had significantly higher odds of anxiety improvement in adjusted models. We detected no significant differences in odds between Gen Xers compared to younger generations (Table 4).

Compared to baseline, 78.39% of Gen Zers and Millennials were no longer reporting moderate to severe depression at 12 weeks vs. 77.76% of Gen Xers, 81.14% of working age Baby Boomers, and 85.51% of retiree age Baby Boomers and Silent Generation adults. Compared to Gen Zers and Millennials, working age Baby Boomers (OR: 1.31, 95% CI: 1.01, 1.71) had significantly higher odds of symptom improvement in adjusted models. We detected no significant differences in odds between Gen Xers or retiree age generations compared to younger generations (Table 4).

## Discussion

This study focused on two objectives. The first objective examined digital MSK program use between generations. Between 86 and 90% of the four generations started the program (i.e., completed one exercise or accessed one education material) after registering.We found that 83% of the retiree age generation completed the program, which exceeded the relatively high completion rates of younger generations (range: 67–82%). In our study, older generations also had more exercise, articles, and messages to coaches compared to younger adults.

Past research suggests some reasons for the increased digital MSK program use among older generations that we observed. First, age interacts with attitude about digital health technology to influence adoption. In paying more attention to their MSK pain, older generations may be more likely than younger adults to use the digital MSK program (27). Second, members of older generations may have appreciated that the programs enabled them to manage their needs themselves and at home, especially among those with mobility or transportation access challenges (16). Third, older generations may have decided to use this technology because they viewed digital health for MSK as being useful and aligned with their needs and values (28). Fourth, support and interaction with live coaches may have further encouraged engagement and helped members to form an exercise habit (29, 30). Evidence suggests that older adults may respond better than younger adults to exercise counseling and education similar to that offered by the program (31).

The second study objective examined change in clinical outcomes among digital MSK program participants, by generation. We did not detect significant differences in changes in pain when comparing older generations to Gen Zers and Millennials. This is in contrast with previous research showing that the benefits of exercise on pain are often more pronounced among younger adults (32). When viewed in conjunction with program use, we interpret our result as showing that older generations need to do more exercise and read more articles to achieve similar changes in pain as younger participants. To better support older adults, future research can examine in greater depth characteristics of older adults (e.g., self-efficacy, environmental factors) who may need to engage more in digital health programs to experience meaningful clinical outcomes.

We also found that older generation was associated with higher odds of anxiety improvement at 12 weeks compared to Gen Zers and Millennials. Previous reviews have shown the effect of 3–12 week exercise programs on improving anxiety, but have found no moderating effect of age (33). The reasons for our program's impact on anxiety among older adults are unclear and warrant additional research. One possibility may be that our program focuses on MSK-related concerns like fall prevention among older adults and addresses anxiety associated with fall-related concerns (34).

Our study participants may not be representative of older adults generally as the study only includes people who opted into a digital MSK program. First, a previous study of a nationally representative sample of older adults found that older adults are less likely to use health information technology vs. younger adults (35). In contrast, our study suggests that older adults who do choose to use a digital MSK program are even more engaged than younger adults. Second, we do not have information about the number eligible for the program or their characteristics. It is not clear how many people were offered the opportunity to participate and if program registration differed by generation. Our program may have included early digital MSK adopters who were more motivated to use or comfortable using technology in daily life. This is in contrast to reports that older adults have less awareness, less trust, lower self-efficacy, and more security and quality concerns about new health technologies (36, 37). Future research can examine self-selection into or out of digital health programs to better tailor programming to later adopters (38). To ensure that digital health programs meet the needs of later adopters, programs should adhere to design best practices that focus on usability and accessibility for older users and persons with disabilities (39).

We examine generational differences in digital MSK program use and outcomes, but generation is a proxy for knowledge, skills, attitudes, and motivations that influence engagement (40). Future research could measure these constructs directly and examine the mediating and moderating effects of age or generation (27).

We use a behavior-based definition of program use that consists of program completion and number of exercises, articles accessed, and coach messages. But these measures may not reflect the “depth” of interaction with the digital MSK program as the measures do not capture affective and cognitive engagement (41). Future research can incorporate broader engagement constructs relevant to older adults and examine the relationship between context, engagement, and behavior change (42).

This study likely omitted important system, predisposing, and enabling factors that influence both program use and health outcomes. For example, the program does not collect predisposing factors such as education or income or enabling factors such as internet access. Further, this prevents us from comparing our study sample to the general adult population to assess generalizability of findings.

This is an observational study that included consecutive program participants meeting inclusion criteria. The large sample sizes in this study may have resulted in detection of spurious relationships between generation and outcomes. In addition, we cannot establish the program's causal effect on pain improvements. However, the results provide evidence about program applicability in the real world with a wide range of ages.

Findings from our study confirm that older generations actively use a digital MSK program that involves app and sensor-guided exercise, app-based education, and remote health coaches. On average, older generations interact with a digital MSK program more than younger counterparts and may experience similar improvements in health outcomes. A digital MSK program holds promise for the growing population of older adults with chronic MSK pain.

## Data Availability Statement

The datasets presented in this article are not readily available because requests for proprietary data will be considered on a case by case basis. Requests to access the datasets should be directed to Grace Wang, grace.wang@hingehealth.com.

## Ethics Statement

The studies involving human participants were reviewed and approved by WCG, Western Institutional Review Board (20160949). Written informed consent for participation was not required for this study in accordance with the national legislation and the institutional requirements.

## Author Contributions

GW and JK contributed to conception and design of the study. GW organized the data. GW, MY, and JB contributed to the statistical analysis. GW wrote the first draft of the manuscript. All authors contributed to manuscript revisions, read, and approved the submitted version.

## Conflict of Interest

GW and JK are employed by and have equity interest in Hinge Health, Inc. JB and MY received consulting fees from Hinge Health, Inc.

## Publisher's Note

All claims expressed in this article are solely those of the authors and do not necessarily represent those of their affiliated organizations, or those of the publisher, the editors and the reviewers. Any product that may be evaluated in this article, or claim that may be made by its manufacturer, is not guaranteed or endorsed by the publisher.

## References

[1] GBD 2019 Diseases and Injuries Collaborators. Global burden of 369 diseases and injuries in 204 countries and territories, 1990-2019: a systematic analysis for the Global Burden of Disease Study 2019. Lancet Lond Engl. (2020) 396:1204–22. 10.1016/S0140-6736(20)30925-9 PMC7567026 33069326

[2] Safiri S Kolahi A-A Smith E Hill C Bettampadi D Mansournia MA. Global, regional and national burden of osteoarthritis 1990-2017: a systematic analysis of the Global Burden of Disease Study 2017. Ann Rheum Dis. (2020) 79:819–28. 10.1136/annrheumdis-2019-216515 32398285

[3] Villarroel M Blackwell D Jen A. Tables of Summary Health Statistics for U.S. Adults: 2018 National Health Interview Survey. National Center for Health Statistics. [Internet]. (2019). Available online at: http://www.cdc.gov/nchs/nhis/SHS/tables.htm (accessed July 15, 2021).

[4] Fonseca-Rodrigues D Rodrigues A Martins T Pinto J Amorim D Almeida A. Correlation between pain severity and levels of anxiety and depression in osteoarthritis patients: a systematic review and meta-analysis. Rheumatol Oxf Engl. (2021) 2021:keab512. 10.1093/rheumatology/keab512 34152386

[5] Bair MJ Robinson RL Katon W Kroenke K. Depression and pain comorbidity: a literature review. Arch Intern Med. (2003) 163:2433–45. 10.1001/archinte.163.20.2433 14609780

[6] Liu F Fang T Zhou F Zhao M Chen M You J. Association of depression/anxiety symptoms with neck pain: a systematic review and meta-analysis of literature in China. Pain Res Manag. (2018) 2018/3259431. 10.1155/2018/3259431 30356353 PMC6176305

[7] Hruschak V Cochran G. Psychosocial predictors in the transition from acute to chronic pain: a systematic review. Psychol Health Med. (2018) 23:1151–67. 10.1080/13548506.2018.1446097 29490476 PMC6708443

[8] Ortego G Villafañe JH Doménech-García V Berjano P Bertozzi L Herrero P. Is there a relationship between psychological stress or anxiety and chronic nonspecific neck-arm pain in adults? A systematic review and meta-analysis. J Psychosom Res. (2016) 90:70–81. 10.1016/j.jpsychores.2016.09.006 27772562

[9] Nicolson PJA Bennell KL Dobson FL Ginckel AV Holden MA Hinman RS. Interventions to increase adherence to therapeutic exercise in older adults with low back pain and/or hip/knee osteoarthritis: a systematic review and meta-analysis. Br J Sports Med. (2017) 51:791–9. 10.1136/bjsports-2016-096458 28087567

[10] Peterson NE Osterloh KD Graff MN. Exercises for older adults with knee and hip pain. J Nurse Pract. (2019) 15:263–67.e3. 10.1016/j.nurpra.2018.12.029

[11] National Guideline Centre (UK). Evidence Review for Exercise for Chronic Primary Pain: Chronic Pain (Primary And Secondary) in Over 16s: Assessment of All Chronic Pain and Management of Chronic Primary Pain: Evidence Review E [Internet]. London: National Institute for Health and Care Excellence (2021). Available online at: http://www.ncbi.nlm.nih.gov/books/NBK569982/33939358

[12] Bridle C Spanjers K Patel S Atherton NM Lamb SE. Effect of exercise on depression severity in older people: systematic review and meta-analysis of randomised controlled trials. Br J Psychiatry J Ment Sci. (2012) 201:180–5. 10.1192/bjp.bp.111.095174 22945926

[13] Siddall B Ram A Jones MD Booth J Perriman D Summers SJ. Short-term impact of combining pain neuroscience education with exercise for chronic musculoskeletal pain: a systematic review and meta-analysis. Pain. (2021). 10.1097/j.pain.0000000000002308. [Epub ahead of print]. 33863860

[14] Snowdon A. Digital Health: A Framework for Healthcare Transformation [Internet]. (2020). Available online at: https://www.himss.org/news/himss-defines-digital-health-global-healthcare-industry (accessed July 15, 2021).

[15] Xie S-H Wang Q Wang L-Q Wang L Song K-P He C-Q. Effect of internet-based rehabilitation programs on improvement of pain and physical function in patients with knee osteoarthritis: systematic review and meta-analysis of randomized controlled trials. J Med Internet Res. (2021) 23:e21542. 10.2196/21542 33399542 PMC7815452

[16] Du S Liu W Cai S Hu Y Dong J. The efficacy of e-health in the self-management of chronic low back pain: a meta-analysis. Int J Nurs Stud. (2020) 106:103507. 10.1016/j.ijnurstu.2019.103507 32320936

[17] Batsis JA DiMilia PR Seo LM Fortuna KL Kennedy MA Blunt HB. Effectiveness of ambulatory telemedicine care in older adults: a systematic review. J Am Geriatr Soc. (2019) 67:1737–49. 10.1111/jgs.15959 31066916 PMC6684409

[18] Greenwald P Stern ME Clark S Sharma R. Older adults and technology: in telehealth, they may not be who you think they are. Int J Emerg Med. (2018) 11:2. 10.1186/s12245-017-0162-7 29299704 PMC5752645

[19] Redfern J. Can older adults benefit from smart devices, wearables, and other digital health options to enhance cardiac rehabilitation? Clin Geriatr Med. (2019) 35:489–97. 10.1016/j.cger.2019.07.004 31543180

[20] Vogels EA. Millennials stand out for their technology use, but older generations also embrace digital life [Internet]. Pew Research Center (2019). Available online at: https://www.pewresearch.org/fact-tank/2019/09/09/us-generations-technology-use/ (accessed Jun 25, 2021).

[21] Tonga E Srikesavan C Williamson E Lamb SE. Components, design and effectiveness of digital physical rehabilitation interventions for older people: a systematic review. J Telemed Telecare. (2020). 10.1177/1357633X20927587. [Epub ahead of print]. 32517544

[22] Andersen RM. Revisiting the behavioral model and access to medical care: does it matter? J Health Soc Behav. (1995) 36:1–10. 10.2307/2137284 7738325

[23] Dimock M. Defining Generations: Where Millennials End and Generation Z Begins [Internet]. Pew Research Center. (2019). Available online at: https://www.pewresearch.org/fact-tank/2019/01/17/where-millennials-end-and-generation-z-begins/ (accessed Jun 25, 2021).

[24] Kroenke K Spitzer RL Williams JBW Löwe B. The patient health questionnaire somatic, anxiety, and depressive symptom scales: a systematic review. Gen Hosp Psychiatry. (2010) 32:345–59. 10.1016/j.genhosppsych.2010.03.006 20633738

[25] Plummer F Manea L Trepel D McMillan D. Screening for anxiety disorders with the GAD-7 and GAD-2: a systematic review and diagnostic metaanalysis. Gen Hosp Psychiatry. (2016) 39:24–31. 10.1016/j.genhosppsych.2015.11.005 26719105

[26] Benjamin S Herr NR McDuffie J Nagi A Williams JW. Performance Characteristics of Self-Report Instruments for Diagnosing Generalized Anxiety and Panic Disorders in Primary Care: A Systematic Review [Internet]. Washington, DC: Department of Veterans Affairs (US) (2011). Available online at: http://www.ncbi.nlm.nih.gov/books/NBK82540/ (accessed June 25, 2021). 22206106

[27] Zhao Y Ni Q Zhou R. What factors influence the mobile health service adoption? A meta-analysis and the moderating role of age. Int J Inf Manag. (2018) 43:342–50. 10.1016/j.ijinfomgt.2017.08.006

[28] Renstrom J. Why Older People Really Eschew Technology. (It's Not Because They Don't Understand It.) [Internet]. Slate Magazine. (2020). Available online at: https://slate.com/technology/2020/07/seniors-technology-illiteracy-misconception-pandemic.html (accessed October 1, 2020).

[29] Brickwood K-J Williams AD Watson G O'Brien J. Older adults' experiences of using a wearable activity tracker with health professional feedback over a 12-month randomised controlled trial. Digit Health. (2020) 6:2055207620921678. 10.1177/2055207620921678 32426152 PMC7218318

[30] Park LG Ng F K Shim J Elnaggar A Villero O. Perceptions and experiences of using mobile technology for medication adherence among older adults with coronary heart disease: a qualitative study. Digit Health. (2020) 6:2055207620926844. 10.1177/2055207620926844 32489672 PMC7241207

[31] Otmanowski JA Chase J-AD. Practical implications for providing physical activity counseling for the older adult: an integrative review. J Am Assoc Nurse Pract. (2020) 32:511–9. 10.1097/JXX.0000000000000483 32658172

[32] Goh SL Persson MSM Stocks J Hou Y Lin J Hall MC. Efficacy and potential determinants of exercise therapy in knee and hip osteoarthritis: a systematic review and meta-analysis. Ann Phys Rehabil Med. (2019) 62:356–65. 10.1016/j.rehab.2019.04.006 31121333 PMC6880792

[33] Herring MP O'Connor PJ Dishman RK. The effect of exercise training on anxiety symptoms among patients: a systematic review. Arch Intern Med. (2010) 170:321–31. 10.1001/archinternmed.2009.530 20177034

[34] Payette M-C Bélanger C Léveillé V Grenier S. Fall-related psychological concerns and anxiety among community-dwelling older adults: systematic review and meta-analysis. PLoS ONE. (2016) 11:e0152848. 10.1371/journal.pone.0152848 27043139 PMC4820267

[35] Onyeaka HK Romero P Healy BC Celano CM. Age differences in the use of health information technology among adults in the united states: an analysis of the health information national trends survey. J Aging Health. (2021) 33:147–54 10.1177/0898264320966266 33031007

[36] Benz J Titus J Malato D Kantor L Sterrett D Alvarez E. Long-Term Care in America: Increasing Access to Care [Internet]. The Long-Term Care Poll. (2018). Available online at: https://www.longtermcarepoll.org/long-term-care-in-america-increasing-access-to-care/ (accessed 2020 August, 21).

[37] Pywell J Vijaykumar S Dodd A Coventry L. Barriers to older adults' uptake of mobile-based mental health interventions. Digit Health. (2020) 6:2055207620905422. 10.1177/2055207620905422 32110429 PMC7016304

[38] Poli A Kelfve S Motel-Klingebiel A. A research tool for measuring non-participation of older people in research on digital health. BMC Public Health. (2019) 19:1487. 10.1186/s12889-019-7830-x 31703655 PMC6842243

[39] Curtis K Price K. Factors that influence older people's engagement with digital health technology. Nurs Older People. (2017) 29:27–30. 10.7748/nop.2017.e986 29188928

[40] O'Connor S Hanlon P O'Donnell CA Garcia S Glanville J Mair FS. Understanding factors affecting patient and public engagement and recruitment to digital health interventions: a systematic review of qualitative studies. BMC Med Inform Decis Mak. (2016) 16:120. 10.1186/s12911-016-0359-3 27630020 PMC5024516

[41] Torous J Michalak EE O'Brien HL. Digital health and engagement-looking behind the measures and methods. JAMA Netw Open. (2020) 3:e2010918. 10.1001/jamanetworkopen.2020.10918 32678446

[42] Perski O Blandford A West R Michie S. Conceptualising engagement with digital behaviour change interventions: a systematic review using principles from critical interpretive synthesis. Transl Behav Med. (2017) 7:254–67. 10.1007/s13142-016-0453-1 27966189 PMC5526809

