# Supplementary figures and images for: Older Adult Use and Outcomes in a Digital Musculoskeletal (MSK) Program, by Generation
Source: Front Digit Health. 2021 Aug 3;3:693170. doi: 10.3389/fdgth.2021.693170 (PMC8521841; doi:10.3389/fdgth.2021.693170)

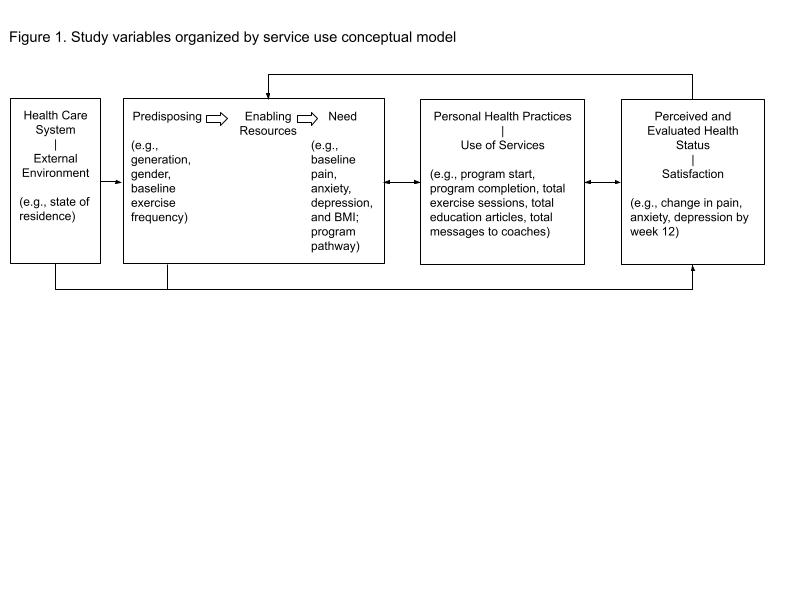

Supplement: Supplementary file 1 [file Image_1.JPEG]
